# Genetic diversity and structure of wild and cultivated *Amorphophallus paeoniifolius* populations in southwestern China as revealed by RAD-seq

**DOI:** 10.1038/s41598-017-14738-6

**Published:** 2017-10-27

**Authors:** Yong Gao, Si Yin, Lifang Wu, Dongqin Dai, Haibo Wang, Chao Liu, Lizhou Tang

**Affiliations:** 1 College of Biological Resource and Food Engineering, Center for Yunnan Plateau Biological Resources Protection and Utilization, Qujing Normal University, Qujing, Yunnan 655011 China; 2 Key Laboratory of Yunnan Province Universities of the Diversity and Ecological Adaptive Evolution for Animals and Plants on YunGui Plateau, Qujing Normal University, Qujing, Yunnan 655011 China; 3 College of Biological Resource and Food Engineering, Qujing Normal University, Qujing, Yunnan 655011 China

## Abstract

*Amorphophallus paeoniifolius*, is a commercially important vegetable crop because of its high production potential. In this study, we generated a total of 166 Gb of genomic data from 16 wild and 20 cultivated *A. paeoniifolius* individuals in southwestern China using restriction site associated DNA sequencing (RAD-seq). We compared the genome-wide variations between the wild and cultivated populations. Wild populations exhibited higher genetic diversity than did cultivated populations based on private allele number, expected heterozygosity, observed heterozygosity and nucleotide diversity. STRUCTURE analysis, principal component analysis (PCA) and a maximum likelihood (ML) tree indicated that *A. paeoniifolius* populations could be divided into three groups (a cultivated group and two wild groups) with significant genetic differentiation. The low genetic diversity and shallow genetic differentiation found within cultivated populations are likely caused by continuous selection and the clonal propagation methods used during domestication. The significant differentiation between the wild populations may suggest strong genetic drift due to small populations and human disturbance. The genome-wide single nucleotide polymorphisms (SNPs) identified in our study will provide a valuable resource for further breeding improvement and effective use of the germplasm.

## Introduction

Crop domestication not only modifies the economic and agronomic traits but also leaves a genetic signature that affects both the genetic diversity and population structure of domesticated plants^1,2^. Accessing genetic variation between and within wild and cultivated populations of crops could provide insight into the general mechanisms of plant domestication and diversification and could guide the genetic improvement of crops in future breeding programs^3,4^.

*Amorphophallus paeoniifolius* (Dennst.) Nicolson is a tropical tuber crop that originates from south-east Asia and belongs to the genus *Amorphophallus*. It is an important economic crop because of its high production potential (50–60 t/ha) and popularity as a vegetable^5^. *Amorphophallus paeoniifolius* is mainly cultivated in India, and serves as an important food resource for humans and as animal feed^6^. Although *Amorphophallus* species have been historically used as a food source and in traditional medicine, *A. paeoniifolius* has not been widely planted in China. However, a new cultivar of *A. paeoniifolius*, *A. paeoniifolius* ‘Yellow’ was discovered in some villages in the southern Yunnan provinces in China in 2012^7^. This cultivar differs from wild *A. paeoniifolius* in several ways, such as its tufty habit, glossy petiole and peduncle^7^. *Amorphophallus paeoniifolius* ‘Yellow’ has been domesticated in these villages for a long time. This cultivar has great potential as a vegetable crop for its fine character, including low fibre content in the corm and good disease resistance^7^.

There is evidence that domestication has led to a reduction in genetic diversity for several cultivated crops^4,8,9^. Genetic information before domestication and artificial selection might have been reserved in wild populations, which are particular resources for studying the influence of human selection on genetic variation in the *A. paeoniifolius* genome. However, the natural populations of *A. paeoniifolius* in China are strongly influenced by human activities, such as harvesting and deforestation, and most remaining populations are restricted to home gardens and agroforestry systems^10^. Thus, conservation measures must be taken to prevent the further decline of *A. paeoniifolius* resources, and information about genetic diversity and population structure is essential for formulating management and conservation approaches. *Amorphophallus paeoniifolius* can outcross, but vegetative propagation is usually used during cultivation and bears significant genetic load. The reliance on clonal propagation and the limited diversity of *A. paeoniifolius* germplasm make it highly vulnerable to many bacterial diseases, such as bacterial soft rot disease^11^. Heredity improvement of *A. paeoniifolius* cultivars using wild germplasms urgently needs to be addressed. Developing genomic resources, increasing understanding of the *A. paeoniifolius* gene pool (including wild germplasms), and gaining information about genetic diversity and population structure should speed the progress of biological research and genetic improvement.

Currently limited genomic information hinders genetic studies of *A. paeoniifolius*. Only a few studies have been carried out to gain information on the genetic diversity of *A. paeoniifolius* and its relationships with relative species using molecular approaches such as simple sequence repeats (SSRs)^12,13^, inter-simple sequence repeats (ISSRs)^14^ and chloroplast DNA loci^15,16^. However, the molecular markers cannot provide sufficient resolution for genetic diversity and genetic structure inferences. Rapid progress in high-throughput sequencing technologies has provided an opportunity to infer genome-wide information from organisms without reference genomes with affordable cost. Reduced-representation genome sequencing allows us to discover thousands of genetic markers from many samples for population genomics studies^17–19^. These methods which consisted of restriction site associated DNA sequencing (RAD-seq) and genotyping by sequencing (GBS) have been successfully applied to population genomics studies of mangy species^20–22^.

To contribute to the understanding of *A. paeoniifolius* domestication and accelerate its agricultural application, we generated and analysed genome-wild SNPs for the wild and cultivated populations of *A. paeoniifolius* (2n = 2x = 28) in southwestern China by RAD-seq to provide a better understanding of the genetic diversity, genetic structure and divergence of this species. Our study will enhance the future genetic improvement of this important crop.

## Results

### Sequence data quality and processing

For the 36 sequenced samples, 166.2 Gb of raw data with an average of 4.49 Gb per sample were generated, ranging from 3.68 to 5.50 Gb. With quality filtering of the sequence data, a total of 161.0 Gb of clean data (3.68 Gb to 5.47 Gb for each sample, with an average of 4.47 Gb) was kept, presenting an average effective rate of 99.56%. In brief, our sequencing data showed high phred quality (Q20 > 90%, Q30 > 85%), with a stable GC content ranging from 41.07% to 44.58% (Table S1).

**Figure 1:**
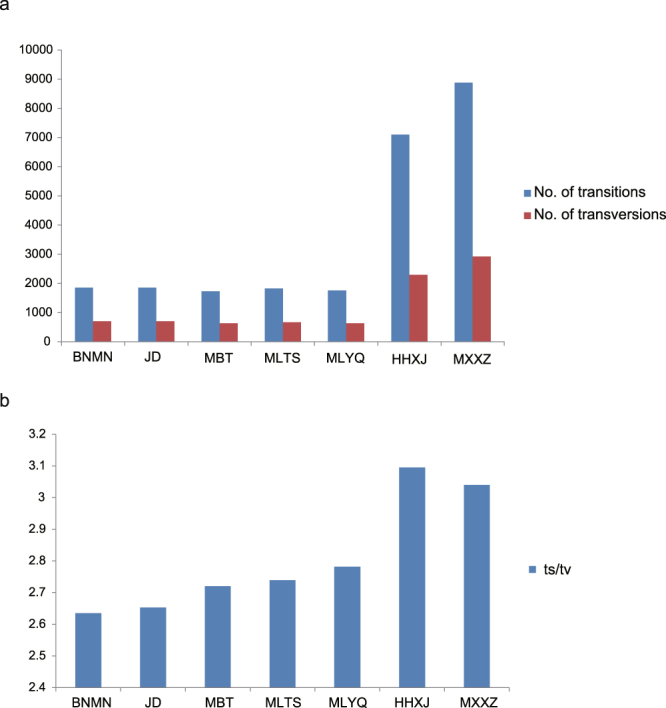
SNP mutation type for the seven populations. (**a**) Number of transitions and transversions for the seven populations. (**b**) Transition/transversion rate.

To access the genetic diversity of *A. paeoniifolius* at the germplasm level (between wild and cultivated germplasms), the 36 samples were divided into two groups. When we required loci to be present in at least 80% of the samples of the two groups, 32,536 RAD loci were retained. When comparing genetic diversity among the seven populations, 19,575 loci were retained after requiring loci to be present in 80% individuals of no less than six populations. More than two thirds of the SNPs at the population level (with an average of 73.70%) were confirmed to be transitions, and the observed transition vs. transversion (ts/tv) ratio ranged from 2.63 to 3.10 for each population. The variation numbers (transitions and transversions) and ts/tv ratios of the SNPs were much higher for the two wild populations of *A. paeoniifolius* than for the populations of *A. paeoniifolius* ‘Yellow’ (Fig. 1, Table S2).

### Genetic diversity at the germplasm and population levels

For all polymorphic loci at the germplasm level, the private allele numbers (*A*_P_), observed heterozygosity (*H*_O_), expected heterozygosity (*H*_E_) and nucleotide diversity (*π*) of the wild germplasm were 25440, 0.2289, 0.3463 and 0.3592, respectively. For *A. paeoniifolius* ‘Yellow’, the *A*_P_, *H*_O_, *H*_E_ and *π* values were 880, 0.1963, 0.1022 and 0.1053, respectively. When analysing all nucleotide positions, including the non-polymorphic sites, the observed heterozygosity, expected heterozygosity and nucleotide diversity of wild *A. paeoniifolius* dropped to 0.0009, 0.0013 and 0.0013, respectively. The three statistics (*H*_O_, *H*_E_ and *π*), including non-polymorphic sites of *A. paeoniifolius* ‘Yellow’, were 0.0007, 0.0004 and 0.0004, respectively (Table 1).

**Table 1:** The statistical values of genetic diversity within populations from variant and all positions data with p = 6/r = 0.8. (*A*_P_, private allele number; *H*_O_, observed heterozygosity; *H*_E_, expected heterozygosity; *π*, nucleotide diversity; *F*_IS_, inbreeding coefficient of an individual relative to the subpopulation).

Taxon	Population	*A*_P_	Polymorphic Loci (%)	*H*_O_		*H*_E_		*π*		*F*_IS_	
				Variant Positions	All Positions	Variant Positions	All Positions	Variant Positions	All Positions	Variant Positions	All Positions
wild *A. paeoniifolius*		25440	0.362	0.2289	0.0009	0.3463	0.0013	0.3592	0.0013	0.3463	0.0012
	MXXZ	8151	0.2021	0.2617	0.0008	0.2517	0.0008	0.2729	0.0008	0.0276	0.0001
	HHXJ	5440	0.2274	0.2584	0.0008	0.2682	0.0008	0.2854	0.0009	0.0634	0.0002
*A. paeoniifolius ‘Yellow'*		880	0.0794	0.1963	0.0007	0.1022	0.0004	0.1053	0.0004	−0.1776	−0.0007
	BNMN	1	0.0425	0.1335	0.0004	0.0668	0.0002	0.1335	0.0004	0	0
	JD	7	0.0431	0.1286	0.0004	0.0663	0.0002	0.0731	0.0002	−0.1011	−0.0003
	MBT	1	0.0414	0.1263	0.0004	0.0644	0.0002	0.0737	0.0002	−0.0923	−0.0003
	MLTS	0	0.0428	0.1264	0.0004	0.0654	0.0002	0.0721	0.0002	−0.099	−0.0003
	MLYQ	5	0.0414	0.1261	0.0004	0.0644	0.0002	0.0773	0.0002	−0.0817	−0.0003

The observed heterozygosity at the population level ranged from 0.1261 (MLYQ) to 0.2617 (MXXZ); the expected heterozygosity for each population ranged from 0.0644 (MBT and MLYQ) to 0.2682 (HHXJ); the nucleotide diversity for each population ranged from 0.0721 (MLTS) to 0.2854 (HHXJ); and the inbreeding coefficient in each population ranged from −0.1011 (JD) to 0.0634 (HHXJ). When considering all nucleotide positions comprising the non-polymorphic sites, the observed heterozygosity dropped to 0.0004 (BNMN, JD, MBT, MLTS and MLYQ) and to 0.0008 (MXXZ and HHXJ); the expected heterozygosity decreased to 0.0002 (BNMN, JD, MBT, MLTS and MLYQ) and to 0.0008 (MXXZ and HHXJ); the nucleotide diversity ranged from 0.0002 (JD, MBT, MLTS and MLYQ) to 0.0009 (HHXJ); and the inbreeding coefficient within each population ranged from −0.0003 (JD, MBT, MLTS and MLYQ) to 0.0002 (HHXJ). The private allele number in each population ranged from 0 (MLTS) to 8151 (MXXZ) (Table 1).

As shown in Table 1, the wild germplasm of *A. paeoniifolius* had much higher genetic diversity than *A. paeoniifolius* ‘Yellow’, which was uncovered by the private allele numbers, the observed heterozygosity, the expected heterozygosity and the nucleotide diversity, regardless of the germplasm or population level.

### Population structure

**Figure 2:**
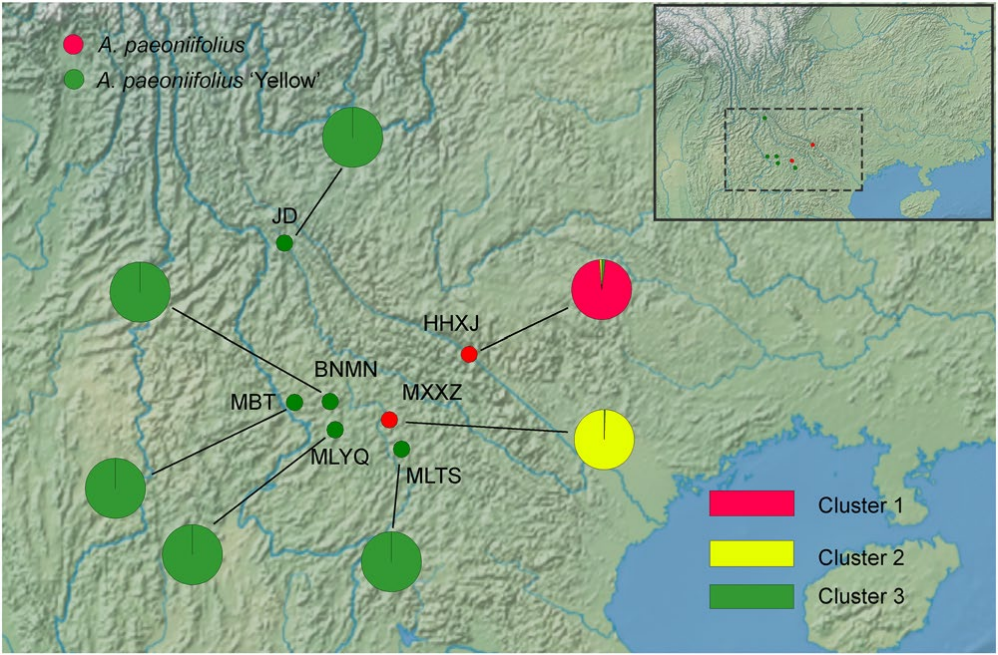
Map of sampling location and results of model-based structure analysis of wild and cultivated *A. paeoniifolius* populations. Individual pie charts indicate mean proportions of membership of each population for the inferred number of *K* = 3 genetic clusters. The map was created with free raster map data from Natural Earth (http://www.naturalearthdata.com/).

**Figure 3:**
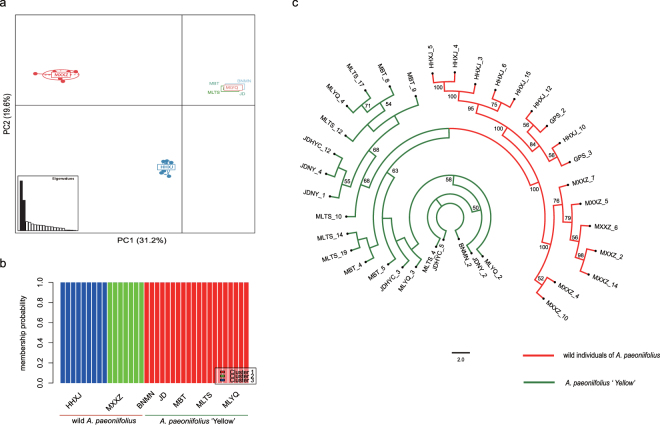
Genome-wide SNP-based genetic structure and phylogeny of wild and cultivated *A. paeoniifolius*. (**a**) Plot of first two dimensions of PCA. (**b**) Bar plot of three clusters identified with R adegenet package. (**c**) Maximum Likelihood phylogram illustrating genetic relationships among 36 individuals. All bootstrap values greater than or equal to 50% are shown.

Genetic analysis of population structure with STRUCTURE software and principal component analysis (PCA) revealed similar patterns. Structure Harvester suggested that *K* = 3 was the most likely genetic cluster number (see Fig. S1). The seven populations were divided into three genetic clusters. Two wild populations of *A. paeoniifolius* were separated into two clusters, and the five populations of *A. paeoniifolius* ‘Yellow’ formed the third genetic cluster (Fig. 2, Fig. 3). Population genetic grouping of *K* = 2 received support as the second highest Δ*K* value. Populations of wild *A. paeoniifolius* and *A. paeoniifolius* ‘Yellow’ formed respective clusters (Fig. S1). The genetic structure was also supported by the results of hierarchical AMOVA: the variance among the three clusters (61.6%) was higher than the genetic variance between wild species and the cultivar (32.4%) (Table 2). Population genetic clustering conformed to that observed in the maximum likelihood (ML) tree constructed with all 36 individuals (Fig. 3c).

**Table 2:** Results of the analyses of molecular variance (AMOVA). Percentage of total genetic variation is given and the corresponding fixation indices are given. (*P < 0.01).

Taxon	Source of variation	Sum of Squares	Variance components	Percentage of variation	Fixation index
Wild species vs. cultivar	Among groups	13206.996	305.479	32.355	*F*_CT_ = 0.324*
	Among populations	10822.458	194.844	20.637	*F*_SC_ = 0.305*
	Within populations	27382.177	443.834	47.008	*F*_ST_ = 0.529*
Three genetic clusters	Among clusters	23992.783	615.697	61.557	*F*_CT_ = 0.616*
	Among populations	36.671	−59.331	−5.932	*F*_SC_ = −0.154*
	Within populations	27382.177	443.834	44.375	*F*_ST_ = 0.556*
Wild *A. paeoniifolius*	Among populations	17844.156	1118.055	44.184	*F*_ST_ = 0.442*
	Within populations	39277.938	1412.393	55.816	—
*A. paeoniifolius* ‘Yellow'	Among populations	119.892	−51.127	−14.737	*F*_ST_ = −0.147
	Within populations	13112.483	398.069	114.737	—

### Genetic differentiation and AMOVA

A moderate level of genetic differentiation (*F*_ST_ = 0.194) was found between wild *A. paeoniifolius* and *A. paeoniifolius* ‘Yellow’ at the germplasm level (*P* < 0.05). The pairwise *F*_ST_ values between populations varied from 0 to 0.2909, with 9 of the 21 population pairs detected with significant values (*P* < 0.05) (Table 3). None of the population pairs in *A. paeoniifolius* ‘Yellow’ were significantly differentiated.

**Table 3:** Pairwise comparison of genetic distances (*F*_ST_ values) (above diagonal) and significance levels (below diagonal) values among seven populations with p = 6/r = 0.8. (*Significance at the 5% nominal level; NS, not significant).

Population	BNMN	HHXJ	JD	MBT	MLTS	MLYQ	MXXZ
BNMN		0.0857	0.0000	0.0000	0.0000	0.0000	0.1111
HHXJ	NS		0.2199	0.1896	0.2193	0.1692	0.2319
JD	NS	*		0.0016	0.0009	0.0021	0.2909
MBT	NS	*	NS		0.0001	0.0000	0.2486
MLTS	NS	*	NS	NS		0.0009	0.2905
MLYQ	NS	*	NS	NS	NS		0.2208
MXXZ	NS	*	*	*	*	*	

The hierarchical analysis of molecular variance (AMOVA) divided the overall genetic variance as 32.4% between the wild and cultivated germplasms, 20.6% among populations within the germplasm and 47.0% within populations (Table 2). AMOVA analysis conducted using the predefined genetic clusters of the population structure analyses suggested that the majority of variance (61.6%) was found among genetic clusters. As the wild germplasm and *A. paeoniifolius* ‘Yellow’ were analysed separately, only the statistics for the wild germplasm were significant, with the variation among populations measured as 44.2% (Table 2).

### Sequence assemble, annotation and GO enrichment analysis

The resultant SNPs consisted of gene-derived (genic) SNPs and non-genic SNPs. Genic SNPs, which represent potential function-related variants, are useful for realizing phenotype mutations, genetic drift and gene flow in cultivated and natural populations; they are especially important for describing genes associated with complex traits^23,24^. To examine genic SNPs, paired-end sequences for each catalogue locus with at least one SNP were assembled. Only contigs with a length of 200 or more nucleotides were recorded for further analyses. Overall, the final assembled sequence comprised of 724783 contigs with an average length of 340 bp and a contig N50 size (50% of the genome is in fragments of this length or longer) of 372 bp (Fig. S2, Table S3).

**Figure 4:**
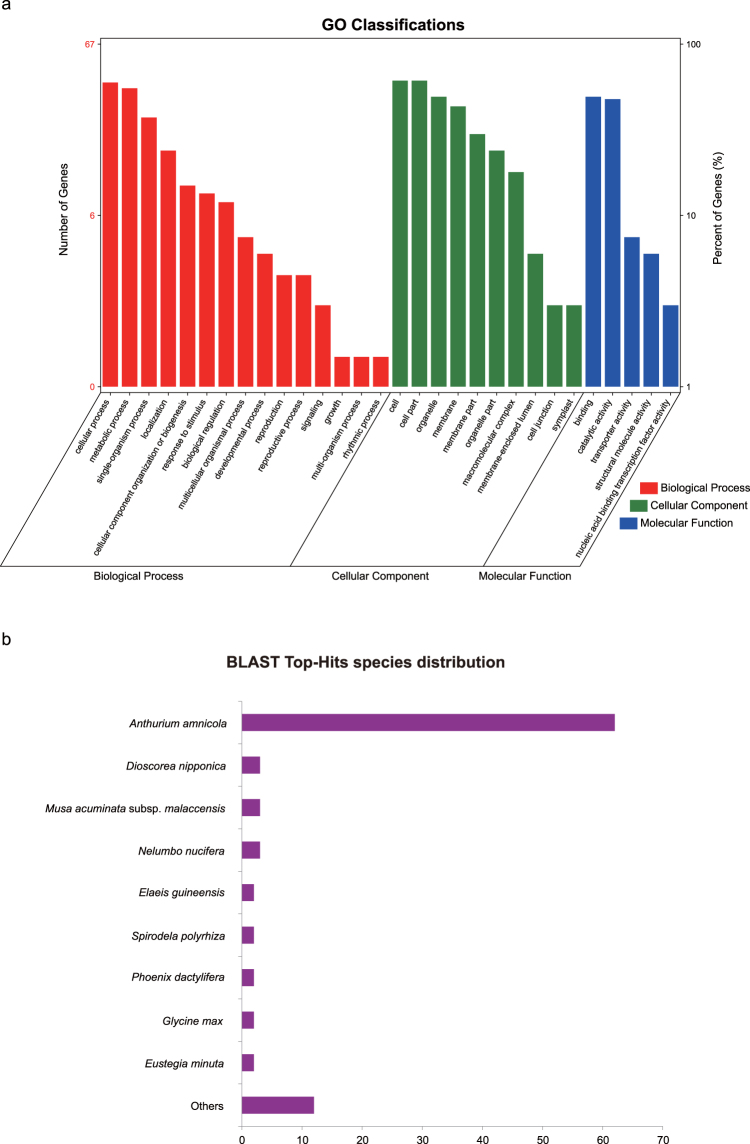
Gene ontology and annotation of genic SNP-associated unigenes. (**a**) GO classifications of identified genic SNP-associated unigenes. (**b**) Frequency and distribution of matched species of significant BLASTX hits (E-value ≤ 1e^−6^).

Based on the assembled transcriptome database of *Amorphophallus konjac*, 107 assembled contigs of *A. paeoniifolius* (0.01%) were aligned to the unigenes of *A. konjac*. According to the annotation results against NCBI’s non-redundant database, of the 107 unigenes that showed sequence similarity with *A. paeoniifolius* sequences, 102 unigenes were significantly mapped to known genes with BLASTX. The annotated sequences were implemented using Blast2GO for GO classification. The top two richest subcategories of the biological processes are metabolic processes and cellular processes. The most highly represented genes under the cellular component category are cell and cell part. Binding and catalytic activity represent the majority of molecular function (Fig. 4, Table S4). The top-hit species distribution inferred by BLAST results is *Anthurium amnicola*, *Dioscorea nipponica*, *Musa acuminate* subsp. *Malaccensis* and *Nelumbo nucifera* (Fig. 4).

## Discussion

We used RAD-seq to assess patterns of genome-wide diversity in the cultivar *A. paeoniifolius* ‘Yellow’ and wild accessions of *A. paeoniifolius* in southwestern China. The analysis revealed that all genetic diversity indices (*H*_O_, *H*_E_ and *π*) for the cultivated group were much lower than the wild accessions across the entire genome when at both the germplasm and population level (Table 1). The excess of rare variants was more significant in wild populations, with 5,440–8,151 private alleles per population, which represented a large gene pool of subsistent genetic variation that could be used in future crop improvement programs. In contrast, only 0–7 private alleles per population were found in the domesticated populations. These differences might be because the continuous selection had reduced effective population size and increased genetic drift and hitchhiking during domestication^2,25^. Inbreeding and intensive selection during domestication, which narrow the germplasm genetic base, reduce the genetic diversity and promote adaptive divergence between domesticated crops, have been reported in many plant species^2,26^.

The number of transitions is predicted be much larger than transversions due to the biased mutational processes within plant genomes (e.g., cytosine deamination). Consistent with this expectation, the ts/tv ratio of the seven *A. paeoniifolius* populations ranged from 2.63 to 3.10. The similar nucleotide mutation pattern was also observed in other plants, such as peanut, maize and *Arabidopsis* ^27–29^. Further analysis also indicted that the ts/tv ratio was higher in wild populations of *A. paeoniifolius* than in cultivated populations. Some researchers claim that transitions are more common than transversions, as they can provide easy tolerance from selection pressure^30^. The relatively low ts/tv ratio in *A. paeoniifolius* ‘Yellow’ might be a loss of evolutionary potential as a consequence of long, severe artificial selection.

Inferring from STRUCTURE analysis, PCA analysis and the ML tree, the seven populations were divided into three genetic clusters. The five populations of *A. paeoniifolius* ‘Yellow’ formed one cluster, and the two wild populations were split into two clusters. The hierarchical AMOVA also revealed a high level of genetic variation among the three clusters (61.6% of the total variation) and a significant (*P* < 0.01) level of variation between the wild germplasms and the cultivar (32.4%) (Table 2). Almost no genetic differentiation was found among the cultivated populations. In most populations of *A. paeoniifolius* ‘Yellow’, farmers brought seeds or tubers from a few individuals from the wild population, and then transferred and grew them in their home gardens. The cultivated populations were then maintained in the village from generation to generation. For most vegetative propagated crops, the separation from wild ancestors during domestication could lower the probabilities of sexual crossing in the subsequent populations^31,32^. Clonal propagation methods may have increased the homogeneity of *A. paeoniifolius* ‘Yellow’ at the population level.

Unlike the cultivated populations, two wild populations showed significant differentiation. The wild accessions of *A. paeoniifolius* in China are distributed over a narrow geographic range (mostly in southern Yunnan province) as comparatively small populations. Human activities in recent years have dramatically influenced the genetic patterns of *A. paeoniifolius* populations, and the disturbances and encroachments from humans are still increasing. Deforestation for construction, farming and grazing has caused continuous and serious damage to the natural populations of *A. paeoniifolius* ^10^. The higher *F*_ST_ values between the two wild populations may suggest stronger genetic drift caused by these factors (Table 3).

We obtained usable information for 102 contigs containing at least one SNP associated with gene function, which might be useful for future studies of the genetic mechanism of the distinct characteristics among wild and cultivated *A. paeoniifolius* plants and the *A. paeoniifolius* genetic breeding program. Although our RAD-seq data consisted of sequences of coding and non-coding regions, and gene annotations for all loci were not assumed, the reason why so few SNPs were annotated (0.01%) is mainly the unavailability of the reference genome data. Although we used the transcriptome dataset from the relative species *A. konjac* ^33^ as the reference database, the genome of *A. paeoniifolius* is the best choice as the reference database to find more comprehensive SNP loci which are related to important agronomic traits such as characteristic secondary metabolism and crop defense of diseases. However, the large genome of *A. paeoniifolius* (proximate 4.21 Gb)^34^ has hindered us to obtain genomic information. In the near future, when the *A. paeoniifolius* plant genome sequencing is complete; we believe that the reference genome data will boost population genetics and functional genetic research in *A. paeoniifolius*.

Populations of *A*. *paeoniifolius* in China have severely declined because of human activities^10^. Although we tried to collect as many samples as possible during field investigation, only 16 wild individuals and 20 cultivated individuals were acquired. It has been shown that using thousands of SNP loci could be powerful for population genetic analysis^35,36^, and the genome-wide SNPs might partly offset limitations due to the small sample size. Nonetheless, the 36 individuals used in our study were not sufficient for assessing the genetic diversity of *A. paeoniifolius* at the whole species level. The cultivar *A. paeoniifolius* ‘Yellow’ is only found in small regions of southern Yunnan province in China^7^. The sample locations in our study represented the full geographical range of the cultivar *A. paeoniifolius* ‘Yellow’. It is believed that this cultivar was domesticated from wild plant materials collected from adjacent areas by farmers^7^. Our sample strategies, which focusing on this small area, should be comprehensive in accessing changes in genetic variation during the domestication of *A. paeoniifolius* ‘Yellow’. Different genetic patterns discovered between wild and cultivated populations in our study could serve as a hint for artificial modifications to the *A. paeoniifolius* genome during domestication in southwestern China. As *A*. *paeoniifolius* has a relatively wide distribution range, further studies with sufficient samples are needed to fully evaluate the genetic diversity of *A. paeoniifolius*.

In conclusion, we reported the exploration of tens of thousands of SNPs to inspect the genetic relationship and compare the genetic diversity of wild and cultivated *A. paeoniifolius* populations by using RAD-seq. We believe this is the first study to report the exploration of such a large number of novel SNPs from *A*. *paeoniifolius* and that further increases the amount of genomic resources available for this species. The results provide new insights into the genetic consequences of crop domestication.

## Methods

### Sample collection and DNA extraction

The materials included 36 samples from 7 populations in south-western China. Specifically, 2 wild populations of *A. paeoniifolius* (16 samples) and 5 populations of the cultivar *A. paeoniifolius* ‘Yellow’ (20 samples) were collected in 2016. A map of the sampling locations was created using software GenGis 2.5.0^37^ (http://kiwi.cs.dal.ca/GenGIS/Main_Page) and free raster map data from Natural Earth (http://www.naturalearthdata.com/) (Fig. 2 and Table S5). Leaves were randomly selected from each population at intervals of at least 5 metres, and were dried in silica gel in sealed plastic bags until DNA extraction. Total genomic DNA was extracted using a Plant Genomic DNA kit (Tiangen, Beijing, China) following the manufacturer’s protocol.

### Creation and sequencing of RAD libraries

RAD sequencing library preparation and sequencing was conducted by the Novogene Bioinformatics Technology CO. ltd, Beijing. Briefly, the libraries were prepared following DNA digestion with *EcoR*I, random fragmentation with the Covaris S220 instrument (Covaris, Woburn, MA, USA), barcode ligation and DNA purification, gel fragment selection, adapter ligation and fragment amplification. Pair-end sequencing with a read length of 150 bp was used to produce approximately 4 Gb of raw data for each sample with the Illumina HiSeq. 2000 platform (Illumina, San Diego, CA, USA).

### De novo assembly and SNP exploitation

The raw data from 36 individuals was first quality-filtered. Adapter sequences and paired reads with alternative reads containing ≥50% low-quality bases (quality value ≤5) or ≥10% unidentified nucleotides were removed. The putative duplication reads and reads without intact *EcoR*I cutting sites were also discarded.

To identify SNP loci for population genetic studies, the Stacks tool set^38^ was utilized. The filtered data for each individual were grouped into loci by ustacks with a minimum stack depth (-m) of 5 and a distance allowed between stacks (-M) of 2. The loci for all samples were then merged into catalogues by cstacks with distances between catalogue loci (-n) of 2, and the loci of each individual were matched against the catalogue to obtain genotypes of the loci in each sample with sstacks.

To analyse the genetic diversity at the germplasm level, the genetic diversity statistics in the two germplasm groups were estimated separately. At least 80% of the samples (r = 0.8) and in all two germplasm groups (p = 2) must contain a locus for it to be included in the analyses. When we analysed the genetic diversity and structure at the population level, a locus was required to be present in 80% of the individuals (r = 0.8) and in no less than six populations (p = 6). Only a single SNP was randomly selected per locus to remove tightly linked SNP loci^39^ in all analyses. Because the sample size in our study was relatively small, SNP loci with a global minor allele frequency (MAF) < 0.05 were therefore discarded to limit false SNP identification. SNP loci were selected using the ‘populations’ program implemented in Stacks^38^. Molecular statistics for the SNPs for the seven populations were analysed using Arlequin 3.5.1^40^.

### Genetic diversity and differentiation

Genetic diversity indices, including the private allele number (*A*_P_), nucleotide diversity (*π*), heterozygosity (*H*_O_ and *H*_E_) and inbreeding coefficient (*F*_IS_), were calculated using the ‘populations’ program in Stacks^38^. To analyse pairwise population differentiation and differentiation between wild and cultivated germplasms, *F*_ST_ values were also computed with the ‘populations’ program and tested based on 1000 permutations with Arlequin 3.5.1^40^.

### Population structure analyses

Genetic structure was investigated by STRUCTURE 2.3.4^41^ and principal component analysis (PCA). The ‘populations’ program in Stacks^38^ was employed to output SNPs into the structure-format file. In the STRUCTURE analysis, an admixture model with correlated allele frequencies between populations was used. Ten replicates for each *K* (*K* = 1–6) were computed, with 50,000 burn-ins followed by 150,000 Markov chain Monte Carlo steps. The optimal *K* was inferred based on the Δ*K* method implemented in STRUCTURE HARVESTER 0.6.94^42^. Admixture proportions from replicate simulations at the optimal *K* were averaged using CLUMPP 1.1.2^43^. Summary outputs were displayed with the program Distruct 2.1^44^. PCA analysis was conducted using the Adegenet package^45^ in R to further investigate the genetic structure between populations. A variant call format (VCF) file was generated using the ‘populations’ program in Stacks^38^. A maximum likelihood (ML) tree of 36 individuals was constructed with 1000 bootstraps using the SNPhylo software^46^.

### AMOVA

AMOVA was conducted to quantify genetic variation at three different hierarchical levels: among germplasm groups, among populations within the germplasm group and within populations. Genetic variations were further tested by assigning populations to genetic clusters identified by population structure analyses (i.e., among genetic clusters, among populations within clusters, within populations). AMOVA was also conducted to assess intra-differentiations within wild or cultivated germplasms. The analyses were conducted in Arlequin 3.5.1^40^, and the significant level for the variance components was computed using 1000 permutation steps.

### Functional analysis of genic SNP-associated genes

To examine genic SNPs, paired-end sequences for each catalogue locus with at least one SNP were collated using the command line ‘sort_read_pairs.pl’ implemented in Stacks^38^. The collated sets of reads for each catalogue locus were then assembled into contigs by the command ‘exec_velvet.pl’, which executes the Velvet^47^ program. To estimate the homology of these contigs to coding regions, a transcriptome dataset of a related species (*A. konjac*) was downloaded from NCBI (SRR555564)^33^ and assembled into unigenes using the Trinity 2.4.0 software^48^. The SNP-associated RAD tag sequences were aligned against the assembled transcriptome of *A. konjac* using SOAP 2.21^49^ with the default settings. To investigate the gene function of loci, a BLASTX search was applied against NCBI’s non-redundant database by BLAST 2.2.28^50^ with an E-value of 1e^−6^. According to the annotation results, Blast2GO 2.5.0^51^ was used to obtain functional classification of the sequences using GO terms with an E-value of 1e^−6^, which maps sequences to gene functions in term of molecular function, cellular components and biological processes^52^.

### Data Availability

The raw RAD sequence data for the 36 wild and cultivated *Amorphophallus paeoniifolius* individuals was deposited in the National Center for Biotechnology Information (NCBI) Sequence-Read Archive (SRA) database with the accession numbers SAMN07175567 - SAMN07175602.

## Electronic supplementary material

Supplementary Figures and Tables
