# Supplementary material for: Genetic diversity and structure of wild and cultivated Amorphophallus paeoniifolius populations in southwestern China as revealed by RAD-seq
Source: Sci Rep. 2017 Oct 27;7:14183. doi: 10.1038/s41598-017-14738-6 (PMC5660214; doi:10.1038/s41598-017-14738-6)
Supplement: Supplementary file 1 — Supplementary Figures and Tables [file 41598_2017_14738_MOESM1_ESM.pdf]

Genetic diversity and structure of wild and cultivated *Amorphophallus paeoniifolius* populations in southwestern China as revealed by RAD-seq

Yong Gao[1,2], Si Yin[3], Lifang Wu[2], Dongqin Dai[1,2], Haibo Wang[1,2], Chao Liu[1,2], Lizhou Tang[1,2*]

[1] College of Biological Resource and Food Engineering, Center for Yunnan Plateau Biological Resources Protection and Utilization, Qujing Normal University, Qujing, Yunnan, 655011, China.

[2] Key Laboratory of Yunnan Province Universities of the Diversity and Ecological Adaptive Evolution for Animals and Plants on YunGui Plateau, Qujing Normal University, Qujing, Yunnan, 655011, China.

[3] College of Biological Resource and Food Engineering, Qujing Normal University, Qujing, Yunnan, 655011, China.

[*]Corresponding author (E-mail: 124472623@qq.com)

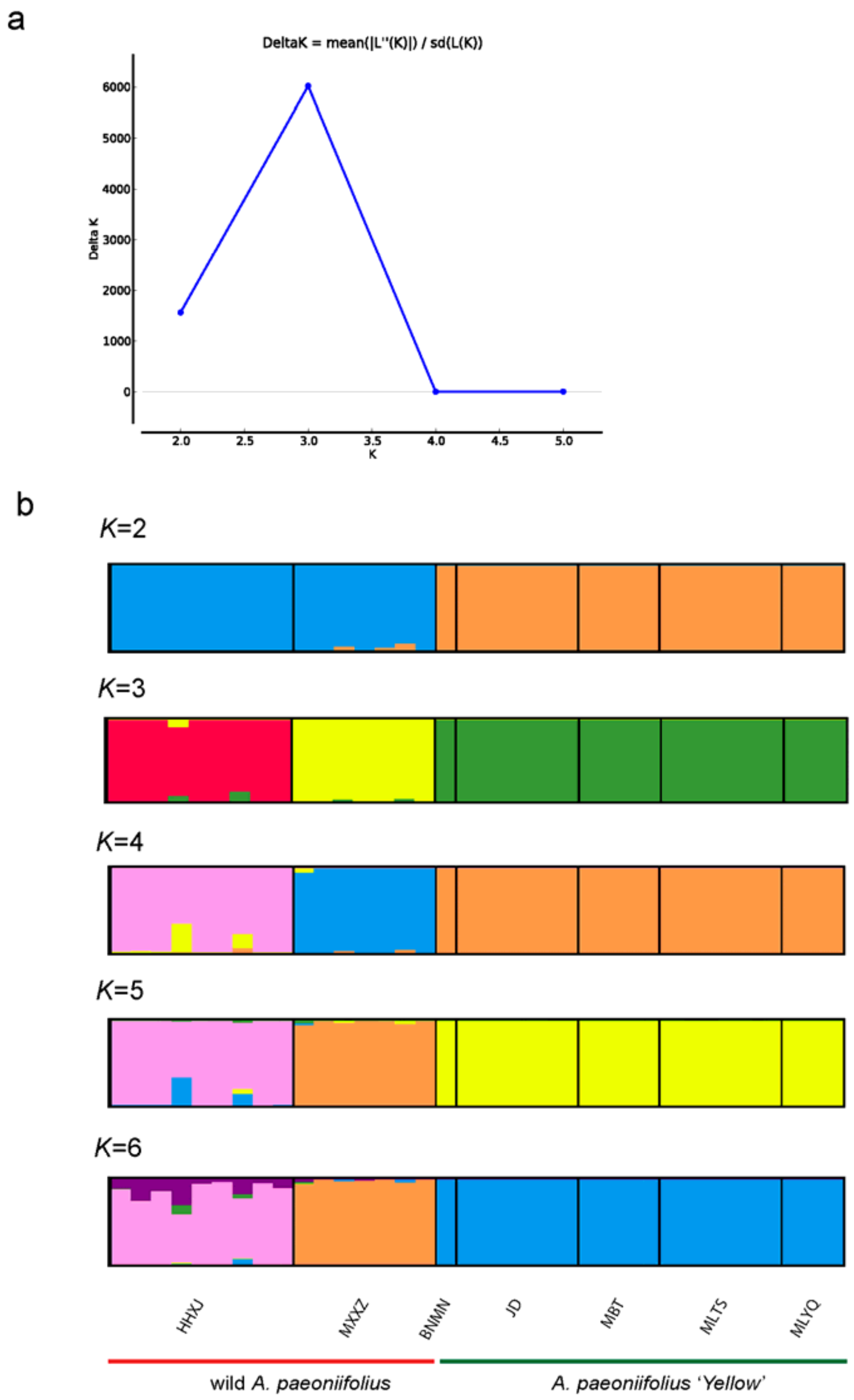

Figure S1. STRUCTURE analysis for the seven wild and cultivated populations. (a) The plots of Δ*K* for each *K* according to Evanno et al. (2005). (b) Population structure estimated using the program STRUCTURE. Each individual is represented by a vertical bar divided into *K* colour segments. The length of each segment is proportional to the estimated value of the membership coefficient (Q).

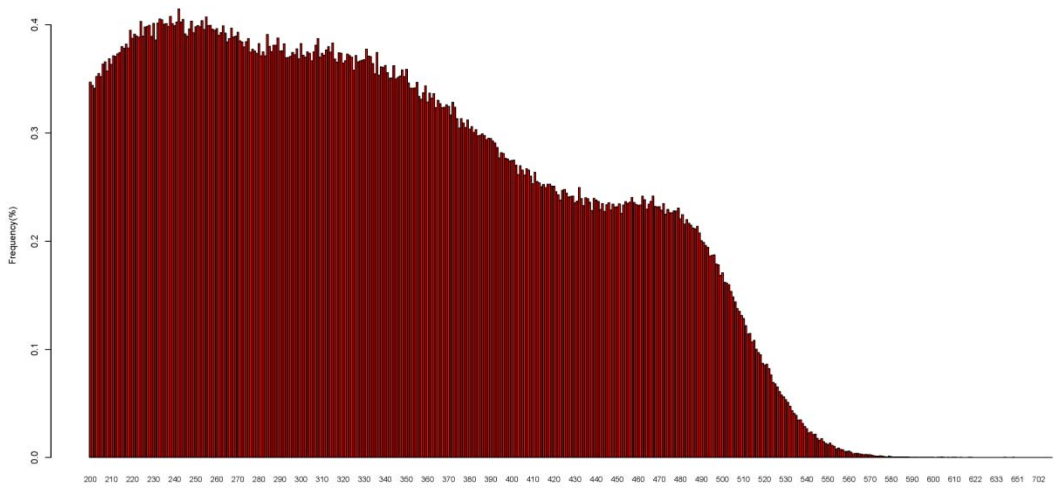

Figure S2. Length distributions of assembled contigs.

Table S1. Summary of the RAD sequencing in the 36 *Amorphophallus paeoniifolius* samples

Sample	Raw Base (Gbp)	Clean Base (Gbp)	Effective Rate (%)	Error Rate (%)	Q20 (%)	Q30 (%)	GC Content (%)
HHXJ-6	4.83	4.82	99.81	0.04	94.50	88.00	44.58
HHXJ-5	3.68	3.68	99.83	0.04	94.69	88.00	43.03
MLTS-12	4.24	4.21	99.47	0.04	95.02	88.00	41.88
MXXZ-7	5.27	5.24	99.45	0.04	95.16	89.00	41.95
MLTS-14	4.15	4.12	99.18	0.04	94.92	88.00	42.25
JDNY-2	5.12	5.11	99.80	0.04	94.67	88.00	41.70
JDNY-1	4.22	4.21	99.69	0.04	94.78	88.00	41.07
GPS-2	5.50	5.47	99.42	0.04	95.63	90.00	42.36
MLTS-17	4.64	4.61	99.31	0.04	95.02	88.00	41.89
MBT-8	4.43	4.42	99.70	0.04	95.01	88.00	42.21
HHXJ-15	5.48	5.45	99.54	0.04	95.38	89.00	41.78
MBT-9	4.82	4.80	99.51	0.04	95.03	89.00	42.09
MXXZ-10	4.86	4.83	99.37	0.04	94.77	88.00	42.08
MLTS-10	4.05	4.02	99.36	0.04	95.44	89.00	42.15
MXXZ-14	4.37	4.34	99.36	0.03	96.12	91.00	41.98
MXXZ-2	5.09	5.08	99.79	0.04	95.30	89.00	42.42
JDHYC-5	4.45	4.44	99.77	0.04	95.09	89.00	42.41
HHXJ-12	4.63	4.61	99.49	0.04	95.58	90.00	42.28
GPS-3	3.93	3.91	99.67	0.04	95.20	89.00	43.00
BNMN-2	4.98	4.95	99.45	0.04	95.41	89.00	42.09
MBT-5	4.58	4.57	99.71	0.03	96.57	92.00	41.89
MBT-4	4.69	4.68	99.70	0.03	96.54	92.00	42.44
MXXZ-6	4.35	4.33	99.52	0.03	96.22	91.00	42.02
MLTS-19	3.75	3.73	99.53	0.03	96.43	91.00	42.26
MLYQ-2	4.21	4.19	99.53	0.03	96.47	92.00	42.07
MLTS-4	4.20	4.17	99.34	0.03	96.14	91.00	42.19
MLYQ-4	4.20	4.18	99.70	0.03	96.73	92.00	42.22
HHXJ-4	4.36	4.35	99.65	0.03	96.00	91.00	42.28
JDNY-4	4.12	4.11	99.63	0.03	95.95	91.00	43.17
HHXJ-3	4.55	4.53	99.75	0.03	96.06	91.00	42.14
MLYQ-3	4.15	4.13	99.64	0.03	96.64	92.00	42.14
MXXZ-5	4.20	4.18	99.42	0.03	96.27	91.00	42.00
MXXZ-4	4.64	4.61	99.31	0.03	95.90	90.00	42.34
HHXJ-10	4.19	4.17	99.40	0.04	94.97	88.00	41.55
JDHYC-3	4.77	4.76	99.68	0.03	96.28	91.00	42.68
JDHYC-12	4.02	4.01	99.72	0.04	95.37	89.00	41.92
Average	4.49	4.47	99.56	0.04	95.59	89.66	42.24
Total	166.20	161.00	-	-	-	-	-

Table S2. The SNP mutation type for the seven populations (ts/tv: transition/transversion rate)

Statistics	BNMN	JD	MBT	MLTS	MLYQ	HHXJ	MXXZ
No.of transitions	1855	1857	1730	1827	1758	7100	8882
No.of transversions	704	700	636	667	632	2294	2922
ts/tv	2.635	2.653	2.720	2.739	2.782	3.095	3.040
transitions rate	0.725	0.726	0.731	0.733	0.736	0.756	0.752

Table S3. Detail information of assmebled contigs with SNPs

Total sequences	Total bases (bp)	Min sequence length	Max sequence length	Average sequence length	N50	(G + C)s
724783	247040308	200	841	340.85	372	42.09%

Table S4. List of the main Gene Ontology categories and sub-categories of the annotated unigenes

Unigene ID	Description	Length	Number of Hits	e-Value	sim mean	Number of GO	GO Names list	Enzyme Codes list	InterPro IDs
TRINITY_DN857_c0_g1_i1	general negative regulator of transcription subunit 4	1264	20	1.580E-127	61.15%	1	F:zinc ion binding		
TRINITY_DN10020_c0_g1_i1	uncharacterized protein loc104602344 isoform x1	919	20	1.220E-101	76.80%	1	F:transferase activity		
TRINITY_DN10636_c0_g1_i1	l-type lectin-domain containing receptor kinase -like	1103	20	5.190E-126	72.05%	3	F:nucleotide binding; C:membrane; F:protein kinase activity		
TRINITY_DN12990_c0_g1_i1	nf-x1-type zinc finger protein nfxl1	818	20	1.600E-141	86.20%	14	F:zinc ion binding; P:response to salt stress; P:regulation of transcription, DNA-templated; P:response to light intensity; P:salicylic acid biosynthetic process; P:regulation of transcription, DNA-templated; P:defense response to bacterium; F:nucleic acid binding; F:nucleotide binding; C:nucleus; C:transcription factor complex; F:transcription factor activity, sequence-specific DNA binding; P:regulation of hydrogen peroxide metabolic process; P:response to microbial		

							phytotoxin
TRINITY_DN13673_c0_g1_i1	autophagy-related protein 18f-like	1724	20	0.000E+00	71.10%	3	P:vesicle-mediated transport; C:cytoplasm; P:response to starvation
TRINITY_DN17660_c0_g1_i1	---NA---	288					
TRINITY_DN18191_c0_g1_i1	nuclear pore complex protein nup133	408	20	4.550E-39	78%	1	C:chloroplast
TRINITY_DN22455_c0_g1_i1	elongator complex protein 1	449	20	1.310E-62	62.50%	7	P:RNA metabolic process; P:regulation of cellular process; P:single-multicellular organism process; P:gene expression; P:response to hormone; C:intracellular part; P:single-organism developmental process
TRINITY_DN23983_c0_g3_i1	protein nuclear fusion defective 4-like	888	20	3.930E-162	80.50%	2	P:transmembrane transport; C:integral component of membrane
TRINITY_DN24410_c0_g2_i1	transforming growth factor-beta receptor-associated protein 1	848	20	2.900E-140	70.40%	1	P:transport
TRINITY_DN25304_c0_g2_i1	uncharacterized protein	1051	20	1.220E-112	76.05%	1	C:membrane

	at1g04910-like						
TRINITY_DN25598_c0_g5_i1	tetratricopeptide repeat-like superfamily protein isoform partial	908	20	1.430E-121	80.65%	1	P:DNA replication-independent nucleosome assembly
TRINITY_DN26381_c0_g1_i1	protein nrt1 ptr family isoform x2	676	20	2.390E-116	83.05%	3	P:oligopeptide transport; C:integral component of membrane; F:transporter activity
TRINITY_DN26517_c0_g2_i1	protein cellulose synthase interactive 1-like	5986	20	0.000E+00	89.55%	5	P:anisotropic cell growth; C:plasmodesma; C:cytoplasm; P:cellulose biosynthetic process; C:plasma membrane
TRINITY_DN26611_c0_g2_i1	npl4-like protein 1	1573	20	1.350E-172	84.50%	4	C:nucleus; F:ubiquitin protein ligase binding; F:ubiquitin binding; P:ubiquitin-dependent protein catabolic process
TRINITY_DN26760_c0_g1_i2	uncharacterized protein loc109825238 isoform x1	858	20	6.110E-122	77.05%	2	C:membrane; C:integral component of membrane
TRINITY_DN27423_c0_g1_i1	importin subunit beta-1-like	1042	20	8.800E-161	86.65%	10	C:nuclear membrane; C:cytoplasm; P:NLS-bearing protein import into nucleus; P:protein import into nucleus, translocation; F:protein transporter activity; C:nuclear periphery; F:Ran GTPase

							binding; P:protein import into nucleus, docking; F:nuclear localization sequence binding; P:ribosomal protein import into nucleus
TRINITY_DN27563_c0_g1_i1	probable polyamine transporter at1g31830 isoform x1	851	20	1.100E-89	88.75%	3	C:integral component of membrane; F:amino acid transmembrane transporter activity; P:amino acid transmembrane transport
TRINITY_DN27895_c0_g1_i2	ribosome maturation protein sbds	1251	20	0.000E+00	87.55%	3	F:nucleic acid binding; F:zinc ion binding; P:mature ribosome assembly
TRINITY_DN28172_c0_g2_i1	pentatricopeptide repeat-containing protein mitochondrial	936	20	6.600E-166	76.30%		
TRINITY_DN28383_c0_g2_i1	peroxisomal membrane protein pex21	681	20	4.130E-60	71.15%		
TRINITY_DN28757_c0_g2_i2	uncharacterized protein loc105034021 isoform x2	1639	20	0.000E+00	78.30%		
TRINITY_DN28804_c1_g1_i3	disease resistance rpp13-like protein 1	727	20	4.480E-39	45.95%	2	F:ADP binding; P:defense response
TRINITY_DN29353_c0_g2_i1	---NA---	536					
TRINITY_DN29420_c0_g1_i1	kh domain-containing protein at4g18375	1403	20	7.950E-149	67.45%	2	F:nucleic acid binding; F:RNA binding

TRINITY_DN29498_c1_g1_i1	fact complex subunit spt16	2137	20	0.000E+00	90.85%	5	C:nucleus; P:DNA repair; P:DNA replication; P:regulation of transcription, DNA-templated; C:chromosome	
TRINITY_DN29743_c0_g3_i3	sensory neuron membrane protein 2	1424	2	1.910E-86	53%			
TRINITY_DN29761_c0_g1_i1	probable membrane metalloprotease chloroplastic	1891	20	5.650E-179	84.45%	3	P:proteolysis; C:integral component of membrane; F:metalloendopeptidase activity	EC:3.4.24.0
TRINITY_DN29761_c0_g1_i2	probable membrane metalloprotease chloroplastic	1944	20	1.000E-178	84.45%	3	P:proteolysis; C:integral component of membrane; F:metalloendopeptidase activity	EC:3.4.24.0
TRINITY_DN29822_c0_g4_i2	serine threonine-protein kinase-like protein cr4	812	20	1.070E-131	88.70%	5	F:ATP binding; C:integral component of membrane; P:serine family amino acid metabolic process; P:protein phosphorylation; F:protein serine/threonine kinase activity	EC:2.7.11.0
TRINITY_DN30153_c0_g3_i1	ft-interacting protein 1-like	2076	20	0.000E+00	94.25%	1	C:integral component of membrane	
TRINITY_DN30297_c1_g3_i1	pre-mrna-splicing factor syf1	835	20	2.310E-137	92.10%	2	C:nucleus; P:mRNA processing	
TRINITY_DN30344_c0_g4_i1	transcriptional adapter ada2	665	20	2.970E-86	74.80%	5	F:DNA binding; C:nucleus; F:zinc ion binding; P:histone acetylation; P:regulation of	

								transcription from RNA polymerase II promoter	
TRINITY_DN30409_c0_g3_i7	---NA---	537							
TRINITY_DN30400_c0_g1_i2	tryptophan synthase beta chain 2	993	20	4.700E-111	82.10%	7	F:tryptophan synthase activity; F:pyridoxal phosphate binding; P:L-phenylalanine biosynthetic process; P:tryptophan biosynthetic process; C:cytoplasm; F:L-serine hydro-lyase (adding indole, L-tryptophan-forming) activity; P:tyrosine biosynthetic process	EC:4.2.1.20	
TRINITY_DN30650_c0_g3_i1	uncharacterized protein loc103994442	2609	20	0.000E+00	61.50%	3	F:ATP binding; F:protein kinase activity; P:protein phosphorylation		
TRINITY_DN30710_c0_g1_i1	pentatricopeptide repeat-containing protein mitochondrial	2289	20	0.000E+00	85.65%	1	C:chloroplast		
TRINITY_DN30722_c0_g4_i1	ribosomal protein l2	1360	20	1.360E-105	95.10%	8	C:chloroplast; F:structural constituent of ribosome; C:mitochondrion; P:ribosome biogenesis; C:large ribosomal subunit; F:transferase activity; F:rRNA binding; P:translation		
TRINITY_DN30722_c0_g6_i3	ribosomal protein s3	1192	20	1.580E-74	97.30%	7	C:chloroplast; F:structural constituent of ribosome;		

							C:mitochondrion; P:ribosome biogenesis; C:small ribosomal subunit; F:rRNA binding; P:translation	
TRINITY_DN30748_c2_g1_i2	cell division cycle 5-like protein	2353	20	0.000E+00	92.75%	2	P:cell division; F:DNA binding	
TRINITY_DN31042_c1_g1_i1	uncharacterized protein loc104603986 isoform x1	3855	20	0.000E+00	66.35%	1	F:nucleotidyltransferase activity	
TRINITY_DN31072_c0_g1_i3	leucine-rich repeat receptor-like tyrosine-protein kinase pxc3	1016	20	1.300E-156	82.85%	4	F:ATP binding; C:integral component of membrane; F:protein kinase activity; P:protein phosphorylation	
TRINITY_DN31139_c2_g2_i1	coatomer subunit alpha-1	2647	20	0.000E+00	96.25%	6	P:vesicle-mediated transport; F:structural molecule activity; C:Golgi membrane; P:intracellular protein transport; F:transferase activity; C:COPI vesicle coat	
TRINITY_DN31165_c0_g1_i2	sucrose synthase 7	1441	20	0.000E+00	88.30%	5	C:cytoplasm; C:membrane; F:sucrose synthase activity; P:starch metabolic process; P:sucrose metabolic process	EC:2.4.1.13
TRINITY_DN31195_c0_g9_i1	probable methyltransferase pmt2	962	20	3.430E-126	89.25%	3	C:integral component of membrane; F:methyltransferase activity; P:methylation	EC:2.1.1.0

TRINITY_DN31318_c0_g2_i7	uncharacterized protein loc104606899 isoform x1	1090	20	1.380E-78	76.35%			
TRINITY_DN31377_c0_g10_i1	1-acyl-sn-glycerol-3-phosphate acyltransferase chloroplastic-like isoform x1	999	20	9.040E-153	80.80%	6	F:1-acylglycerol-3-phosphate O-acyltransferase activity; P:acyl-carrier-protein biosynthetic process; P:embryo development ending in seed dormancy; C:integral component of membrane; C:chloroplast envelope; P:phosphatidylglycerol biosynthetic process	EC:2.3.1.51
TRINITY_DN31370_c0_g1_i1	serine threonine-protein kinase edr1 isoform x1	1653	20	0.000E+00	75.40%	2	F:protein kinase activity; P:phosphorylation	
TRINITY_DN31383_c0_g3_i2	btb poz domain-containing protein at1g63850-like	2708	20	0.000E+00	84.50%	1	C:plastid	
TRINITY_DN31385_c1_g1_i1	protein disulfide-isomerase	1056	20	6.210E-161	64.75%	4	F:nucleic acid binding; F:ATP binding; F:helicase activity; F:hydrolase activity	
TRINITY_DN31405_c0_g4_i1	dna damage-binding protein 1a	356	20	1.360E-76	99.30%	5	C:nucleus; P:proteasome-mediated ubiquitin-dependent protein catabolic process; C:cullin-RING ubiquitin ligase complex; P:nucleotide-excision repair; F:damaged DNA binding	

TRINITY_DN31534_c2_g6_i2	protein basic pentacysteine7	1422	20	1.230E-151	71.15%	1	C:nucleus
TRINITY_DN31548_c2_g4_i1	probable lrr receptor-like serine threonine-protein kinase at4g20940 isoform x2	652	20	7.620E-123	97.05%	3	F:ATP binding; F:protein kinase activity; P:protein phosphorylation
TRINITY_DN31549_c1_g4_i3	trihelix transcription factor asil2-like	1720	20	2.620E-108	76.90%	1	F:DNA binding
TRINITY_DN31576_c2_g1_i1	50s ribosomal protein partial	1145	20	1.750E-40	83.70%	5	F:structural constituent of ribosome; C:mitochondrion; P:ribosome biogenesis; C:ribosome; P:translation
TRINITY_DN31596_c1_g3_i1	swi snf complex component snf12 homolog	798	20	8.860E-179	91.60%		
TRINITY_DN31674_c0_g2_i2	subfamily c member 7	1671	20	5.790E-172	69.55%		
TRINITY_DN31755_c0_g2_i4	translation initiation factor if-partial	1882	20	0.000E+00	85.05%		
TRINITY_DN31827_c2_g1_i5	protein cdc73 homolog	1433	20	0.000E+00	74.60%	4	C:nucleus; P:reproductive structure development; P:histone modification; P:post-embryonic development
TRINITY_DN31863_c2_g1_i3	uncharacterized protein loc102595024 isoform x1	1198	20	3.360E-83	71.25%	2	F:nucleic acid binding; F:zinc ion binding
TRINITY_DN31907_c0_g5_i1	protein iq-domain 32-like	838	20	1.620E-74	65.75%		
TRINITY_DN31926_c1_g2_i1	hypothetical chloroplast rf21	1861	20	1.720E-97	92.90%	3	C:chloroplast stroma; F:ATP binding; C:integral component of membrane

TRINITY_DN31926_c1_g2_i6	ribulose- -biphosphate carboxylase oxygenase large partial	2100	20	1.810E-169	99%	6	F:magnesium ion binding; C:chloroplast ribulose bisphosphate carboxylase complex; P:glyoxylate metabolic process; P:photosynthesis; P:carbon fixation; F:ribulose-bisphosphate carboxylase activity	EC:4.1.1.39
TRINITY_DN32099_c0_g1_i3	retrovirus-related pol polyprotein from transposon tnt 1-94	1510	20	1.830E-97	55.45%	5	F:nucleic acid binding; F:zinc ion binding; C:membrane; C:integral component of membrane; P:DNA integration	
TRINITY_DN32067_c2_g3_i1	rna polymerase beta subunit	950	20	0.000E+00	96.20%	7	F:DNA binding; C:chloroplast; P:pyrimidine nucleobase metabolic process; F:DNA-directed RNA polymerase activity; P:transcription, DNA-templated; P:purine nucleobase metabolic process; C:nucleolus	EC:2.7.7.6
TRINITY_DN32067_c2_g4_i1	rna polymerase beta subunit	949	20	0.000E+00	96.15%	8	F:DNA binding; C:chloroplast; P:pyrimidine nucleobase metabolic process; F:DNA-directed RNA	EC:2.7.7.6

							polymerase activity; F:ribonucleoside binding; P:transcription, DNA-templated; P:purine nucleobase metabolic process; C:nucleolus	
TRINITY_DN32079_c2_g4_i1	nadh-plastoquinone oxidoreductase subunit partial	712	20	5.290E-121	96.05%	8	P:sodium ion transport; C:chloroplast; P:proton transport; F:quinone binding; P:mitochondrial electron transport, NADH to ubiquinone; C:integral component of membrane; F:NADH dehydrogenase (ubiquinone) activity; P:ubiquinone biosynthetic process	EC:1.6.5.3
TRINITY_DN32136_c5_g1_i1	wd40-like beta propeller	3038	20	0.000E+00	73.85%			
TRINITY_DN32118_c1_g1_i4	uncharacterized protein loc103992931	941	20	5.810E-29	74.35%			
TRINITY_DN32175_c0_g1_i1	hypothetical protein GLYMA_13G021700, partial [Glycine max]	3147	20	0.000E+00	96.35%			
TRINITY_DN32175_c0_g2_i1	uncharacterized protein partial	632	20	6.750E-62	94.45%	2	P:nucleic acid phosphodiester bond hydrolysis; F:endonuclease activity	

TRINITY_DN32175_c0_g1_i6	hypothetical protein GLYMA_13G021700, partial [Glycine max]	3526	20	0.000E+00	96.35%		
TRINITY_DN32176_c3_g6_i1	uncharacterized protein loc107643668	1230	20	4.510E-60	88.10%	3	C:chloroplast; F:metal ion binding; F:catalytic activity
TRINITY_DN32178_c4_g1_i3	ft-interacting protein 1-like	1538	20	1.050E-177	68.35%	3	F:calcium ion binding; C:plasma membrane; F:calcium-dependent phospholipid binding
TRINITY_DN32236_c1_g8_i2	---NA---	773					
TRINITY_DN32217_c2_g1_i2	eukaryotic translation initiation factor 3 subunit c	3384	20	0.000E+00	83.25%	8	F:translation initiation factor activity; P:formation of translation preinitiation complex; C:eukaryotic 43S preinitiation complex; C:eukaryotic 48S preinitiation complex; P:regulation of translational initiation; F:translation initiation factor binding; C:ribosome; C:eukaryotic translation initiation factor 3 complex

TRINITY_DN32308_c5_g1_i2	della protein gai-like	2538	20	0.000E+00	69.35%	15	P:hyperosmotic salinity response; P:negative regulation of seed germination; P:regulation of transcription, DNA-templated; P:salicylic acid mediated signaling pathway; F:sequence-specific DNA binding; P:regulation of transcription, DNA-templated; P:jasmonic acid mediated signaling pathway; P:response to ethylene; P:negative regulation of gibberellic acid mediated signaling pathway; C:nucleus; C:transcription factor complex; F:transcription factor activity, sequence-specific DNA binding; P:response to abscisic acid; P:regulation of reactive oxygen species metabolic process; P:regulation of seed dormancy process
TRINITY_DN32313_c0_g1_i2	transport inhibitor response 1-like protein os04g0395600	692	20	3.160E-58	87.70%	6	C:nucleus; F:auxin binding; F:inositol hexakisphosphate binding; C:SCF ubiquitin ligase

							complex; P:auxin-activated signaling pathway; P:protein ubiquitination	
TRINITY_DN32322_c2_g11_i1	nadh dehydrogenase subunit 7	2303	20	0.000E+00	97.05%	9	F:NADH dehydrogenase (quinone) activity; P:obsolete electron transport; F:quinone binding; F:NAD binding; P:photosynthesis, light reaction; P:oxidation-reduction process; P:transport; C:plasma membrane; C:chloroplast thylakoid membrane	EC:1.6.99.5
TRINITY_DN32322_c2_g11_i2	nadh dehydrogenase subunit 7	2567	20	0.000E+00	97.05%	9	F:NADH dehydrogenase (quinone) activity; P:obsolete electron transport; F:quinone binding; F:NAD binding; P:photosynthesis, light reaction; P:oxidation-reduction process; P:transport; C:plasma membrane; C:chloroplast thylakoid membrane	EC:1.6.99.5
TRINITY_DN32331_c0_g1_i4	dnaj protein erdj3b-like	3051	20	0.000E+00	92.50%	2	F:unfolded protein binding; P:protein folding	

TRINITY_DN32355_c1_g2_i3	abc transporter b family member 1	3091	20	0.000E+00	94.15%	17	F:ATPase activity, coupled to transmembrane movement of substances; P:transmembrane transport; P:photomorphogenesis; P:regulation of cell size; P:response to blue light; C:plasma membrane; P:auxin influx; P:auxin polar transport; F:ATP binding; P:positive gravitropism; C:plasmodesma; F:auxin efflux transmembrane transporter activity; F:auxin influx transmembrane transporter activity; P:anthocyanin accumulation in tissues in response to UV light; P:stamen development; C:integral component of membrane; P:auxin efflux
TRINITY_DN32366_c0_g1_i1	dna polymerase i chloroplastic mitochondrial	2665	20	0.000E+00	81.05%		
TRINITY_DN32534_c0_g3_i1	basic 7s globulin 2-like	1615	20	3.430E-128	63.05%	3	P:protein metabolic process; P:macromolecule catabolic process; F:hydrolase activity

TRINITY_DN32543_c3_g1_i1	metallocarboxypeptidase inhibitor	2456	20	5.190E-93	88.15%	2	C:chloroplast; C:mitochondrion	
TRINITY_DN32615_c0_g2_i9	elongator complex protein 4	567	20	1.760E-12	84.15%	1	C:Elongator holoenzyme complex	
TRINITY_DN32604_c0_g1_i1	nadh-plastoquinone oxidoreductase subunit 2	2370	20	0.000E+00	97.40%	10	P:sodium ion transport; P:proton transport; F:quinone binding; P:mitochondrial electron transport, NADH to ubiquinone; C:integral component of membrane; F:NADH dehydrogenase (ubiquinone) activity; P:photosynthesis, light reaction; P:ubiquinone biosynthetic process; C:plasma membrane; C:chloroplast thylakoid membrane	EC:1.6.5.3
TRINITY_DN32604_c0_g1_i3	nadh dehydrogenase subunit 2	4782	20	1.220E-170	92.50%	10	P:sodium ion transport; P:proton transport; F:quinone binding; P:mitochondrial electron transport, NADH to ubiquinone; C:integral component of membrane; F:NADH dehydrogenase (ubiquinone) activity; P:photosynthesis, light reaction;	EC:1.6.5.3

							P:ubiquinone biosynthetic process; C:plasma membrane; C:chloroplast thylakoid membrane	
TRINITY_DN32604_c0_g1_i4	nadh dehydrogenase subunit 2	4681	20	6.800E-171	92.50%	10	P:sodium ion transport; P:proton transport; F:quinone binding; P:mitochondrial electron transport, NADH to ubiquinone; C:integral component of membrane; F:NADH dehydrogenase (ubiquinone) activity; P:photosynthesis, light reaction; P:ubiquinone biosynthetic process; C:plasma membrane; C:chloroplast thylakoid membrane	EC:1.6.5.3
TRINITY_DN32604_c0_g1_i5	nadh dehydrogenase subunit 2	4432	20	1.530E-171	92.50%	10	P:sodium ion transport; P:proton transport; F:quinone binding; P:mitochondrial electron transport, NADH to ubiquinone; C:integral component of membrane; F:NADH dehydrogenase (ubiquinone) activity; P:photosynthesis, light reaction;	EC:1.6.5.3

							P:ubiquinone biosynthetic process; C:plasma membrane; C:chloroplast thylakoid membrane
TRINITY_DN32619_c1_g4_i5	oxygen-evolving enhancer protein 3- chloroplastic	2621	20	3.140E-51	69.35%	1	C:membrane part
TRINITY_DN32679_c4_g5_i1	mediator of rna polymerase ii transcription subunit 16 isoform x1	1282	20	0.000E+00	88.25%	8	P:circadian regulation of gene expression; P:regulation of long-day photoperiodism, flowering; C:mediator complex; P:positive regulation of systemic acquired resistance; P:response to osmotic stress; P:regulation of ethylene-activated signaling pathway; P:regulation of transcription, DNA-templated; P:regulation of jasmonic acid mediated signaling pathway
TRINITY_DN32715_c1_g2_i2	subtilisin-like protease	1840	20	0.000E+00	67.90%	1	F:peptidase activity
TRINITY_DN32841_c1_g2_i4	lrr receptor-like serine threonine-protein kinase fls2	1703	20	1.460E-164	71.90%	4	F:oxidoreductase activity; C:membrane; P:phosphorylation; F:kinase activity
TRINITY_DN32836_c1_g1_i10	abc transporter c family member 15	2242	20	5.010E-130	89.05%	4	F:ATP binding; F:ATPase activity, coupled to

							transmembrane movement of substances; P:transmembrane transport; C:integral component of membrane
TRINITY_DN32927_c0_g1_i1	u-box domain-containing protein 19-like	1039	20	3.480E-160	69.05%	2	F:ubiquitin-protein transferase activity; P:protein ubiquitination
TRINITY_DN46000_c0_g1_i1	4-coumarate-- ligase-like 5	516	20	2.500E-37	72.05%	1	F:catalytic activity

Table S5. Characteristics of 36 *A. paeoniifolius* and *A. paeoniifolius* 'Yellow' samples in this study

Population Id	Taxon	Sample Size	Latitude	Longitude	Site
MXXZ	wild *A. paeoniifolius*	7	21.878	102.363	Xishuangbanna, Yunnan
HHXJ	wild *A. paeoniifolius*	9	22.868	103.568	Honghe, Yunnan
JD	*A. paeoniifolius* 'Yellow'	6	24.542	100.779	Simao, Yunnan
MBT	*A. paeoniifolius* 'Yellow'	4	22.141	100.930	Xishuangbanna, Yunnan
MLTS	*A. paeoniifolius* 'Yellow'	6	21.434	102.548	Xishuangbanna, Yunnan
MLYQ	*A. paeoniifolius* 'Yellow'	3	21.727	101.544	Xishuangbanna, Yunnan
BNMN	*A. paeoniifolius* 'Yellow'	1	22.153	101.469	Xishuangbanna, Yunnan
